# Dysregulation of the Immune System in Advanced Periimplantitis: Systemic Implications and Inflammatory Mechanisms—A Hematological and Immunological Study

**DOI:** 10.3390/jcm14072453

**Published:** 2025-04-03

**Authors:** Michał Łobacz, Mansur Rahnama-Hezavah, Paulina Mertowska, Sebastian Mertowski, Katarzyna Wieczorek, Grzegorz Hajduk, Ewelina Grywalska

**Affiliations:** 1 Chair and Department of Oral Surgery, Medical University of Lublin, 20-093 Lublin, Poland; mansur.rahnama-hezavah@umlub.pl (M.R.-H.); katwieczorek@wp.pl (K.W.); esso53@wp.pl (G.H.); 2 Department of Experimental Immunology, Medical University of Lublin, 20-093 Lublin, Poland; paulinamertowska@umlub.pl (P.M.); sebastian.mertowski@umlub.pl (S.M.); ewelina.grywalska@umlub.pl (E.G.)

**Keywords:** periimplantitis, inflammatory markers, immune dysregulation, T-cell exhaustion, PD-1, PD-L1, natural killer (NK) cells, systemic inflammation, leukocyte count, immunomodulation

## Abstract

**Objectives:** This study aimed to assess the systemic and local inflammatory responses in patients with periimplantitis, focusing on key immune markers and clinical parameters. The study further explores the relationship between inflammatory markers, clinical indices, and immune dysregulation, particularly regarding T-cell exhaustion and systemic inflammation. **Methods:** A cohort of patients with periimplantitis, classified into moderate and advanced stages, was compared to a control group of healthy individuals with dental implants. Clinical parameters, including plaque index (API), bleeding on probing (BoP), probing pocket depth (PPD), and peri-implant sulcus depth (PSI), were recorded. Hematological, immunological, and biochemical analyses were performed, with a focus on immune cell populations (NK cells, T-cells, and their exhaustion markers PD-1 and PD-L1). **Results:** Patients with periimplantitis exhibited significantly higher clinical indices (API, BoP, PSI, and PPD) than the control group, with the most pronounced differences in the advanced periimplantitis group. Hematological analysis revealed increased leukocyte and neutrophil counts, whereas NK cell levels were significantly reduced. Immunological profiling indicated elevated PD-1 and PD-L1 expression on T-cells, suggesting T-cell exhaustion and immune dysregulation. Furthermore, strong correlations were found between increased PPD values and elevated inflammatory marker levels, highlighting the relationship between peri-implant pocket depth and systemic inflammation. **Conclusions:** The findings confirm that immune dysregulation plays a central role in periimplantitis progression. The association between increased inflammatory markers, immune alterations, and clinical indices emphasizes the need for a multifactorial diagnostic and treatment approach. Integrating immune modulation strategies, clinical assessments, and lifestyle modifications, such as improved oral hygiene and smoking cessation, could improve disease management and reduce recurrence.

## 1. Introduction

Periimplantitis is a chronic inflammatory disease that leads to the destruction of soft tissues and bone loss around dental implants, which may ultimately result in implant failure. Its pathogenesis is driven by both microbial dysbiosis and excessive host immune response, including uncontrolled activation of the immune system [1,2,3]. Similar to periodontitis, inflammation in periimplantitis is associated with elevated levels of pro-inflammatory cytokines such as interleukin-1β (IL-1β), interleukin-6 (IL-6), and tumor necrosis factor-alpha (TNF-α) [4,5]. However, recent studies suggest that immune dysregulation, involving both local and systemic immune mechanisms, plays a crucial role in disease progression [6,7].

One of the key aspects of the immune response in periimplantitis is the role of T lymphocytes and NK cells, as well as their functional exhaustion. T-cell exhaustion, characterized by the overexpression of inhibitory receptors such as PD-1 and its ligand PD-L1, is a well-known immunosuppressive mechanism in chronic infections and inflammatory diseases [8,9]. The expression of these markers in periimplantitis suggests that chronic inflammation may impair the function of key immune cells, contributing to disease progression and a weakened ability to control infection [10,11]. Previous studies indicate that patients with periimplantitis exhibit a reduced number of NK cells and cytotoxic CD8+ T lymphocytes, which may suggest their functional exhaustion [12,13]. Beyond local changes, periimplantitis may also influence systemic inflammation. Studies have shown that patients with periimplantitis have elevated levels of inflammatory markers such as C-reactive protein (CRP) and IL-6, which may increase the risk of developing systemic diseases, including cardiovascular and metabolic disorders [14,15,16]. Understanding these mechanisms is essential for developing effective therapeutic strategies that not only control local inflammation but also minimize its systemic consequences [17,18]. Although numerous studies have focused on the microbiological and clinical aspects of periimplantitis, there is a lack of detailed analysis of the immunological mechanisms contributing to the chronicity of the disease. In particular, the role of T-cell exhaustion and immunosuppression associated with PD-1 and PD-L1 expression remains insufficiently explored [19,20,21]. Moreover, it is still unclear to what extent these immunological changes correlate with disease severity and whether they hold diagnostic or therapeutic significance [22,23]. This study aims to evaluate the role of immune dysregulation in periimplantitis, with a specific focus on T-cell exhaustion and PD-1/PD-L1 expression. The analysis includes both local immune responses in peri-implant tissues and systemic changes observed in peripheral blood. The following key questions were addressed in this study: assessment of inflammatory marker levels and immune cell populations in patients with periimplantitis compared to a control group [24,25]; analysis of PD-1 and PD-L1 expression on T and NK cells and their potential role in immunosuppression and disease progression [26]; determination of the correlation between clinical severity (peri-implant pocket depth) and systemic immune alterations [27]; and identification of potential diagnostic and therapeutic targets for immune modulation in periimplantitis [28]. The findings of this study may contribute to a better understanding of periimplantitis as an immunologically driven disease rather than merely a localized infectious process. Identifying biomarkers related to immunosuppression could open new therapeutic opportunities focused on modulating immune responses to slow disease progression and reduce the risk of recurrence.

## 2. Materials and Methods

This study is a cross-sectional observational study designed to assess immune dysregulation in patients with periimplantitis. The study included 74 participants, divided into a study group consisting of 50 patients with dental implants and a control group of 20 individuals without implants or oral health issues. The study group was further categorized into three subgroups based on the severity of periimplant disease: The first subgroup included 18 patients with advanced periimplantitis, characterized by severe peri-implant bone loss, probing depths exceeding 6 mm, bleeding on probing, and radiographic evidence of progressive bone destruction. The mean age in this subgroup was 47.2 years (range: 41–60), with a gender distribution of 8 females and 10 males. The second subgroup comprised 16 patients with moderate periimplantitis, who exhibited moderate peri-implant bone loss, peri-implant pockets of 4–6 mm, and clinical signs of inflammation. The mean age in this subgroup was 48.4 years (range: 41–60), with 7 females and 9 males. The third subgroup consisted of 16 patients with dental implants but no clinical or radiographic signs of periimplantitis, serving as a control group within the implant-bearing population. These individuals had stable peri-implant tissues without signs of inflammation or bone resorption. The mean age in this subgroup was 48.3 years (range: 41–60), with 7 females and 9 males. The control group, consisting of 20 individuals without dental implants or any oral health issues, served as a baseline reference for systemic inflammatory and immune parameters. The mean age in this group was 48.1 years (range: 41–60), with an equal gender distribution of 10 females and 10 males. The sample size was determined based on statistical power considerations and feasibility constraints. A preliminary power analysis indicated that a minimum of 15 patients per group would be required to detect significant differences in immunological markers with an effect size of 0.8, a power of 80%, and a significance level of α = 0.05. The slight variation in group sizes reflects the availability of eligible participants within the study period. All patients in the study group had two-stage titanium dental implants from various manufacturers, ensuring a representative sample of commonly used implant systems. The inclusion of different implant types enhances the generalizability of findings, as periimplantitis can affect implants with different surface properties and macrodesigns.

The selection of group sizes was based on a balance between statistical requirements and feasibility, ensuring sufficient data for meaningful analysis while maintaining practical recruitment constraints. The demographic characteristics, including age distribution, gender ratio, and implant types, were documented to ensure transparency and enable generalization of the results to a broader population. All participants provided informed consent before enrollment, agreeing to clinical assessments and blood sample collection. Exclusion criteria included metabolic diseases such as diabetes mellitus, osteoporosis, or osteomalacia, as well as smoking and recent antibiotic or anti-inflammatory treatments. Additionally, individuals with autoimmune or chronic inflammatory diseases and pregnant or lactating women were excluded.

The clinical evaluation included a comprehensive oral examination, including a medical history, clinical evaluation, a panoramic X-ray, and periodontal status analysis. The following parameters were recorded as part of the clinical evaluation:

- API (Approximal Plaque Index)—determining the degree of plaque accumulation around implants,
- BoP (Bleeding on Probing)—assessing the inflammation of peri-implant tissues based on bleeding on probing,
- PSI (Periodontal Screening Index)—determining the general health of the periodontium,
- PPD (Probing Pocket Depth)—measuring the depth of peri-implant pockets.

The methods for recording clinical parameters (API, BoP, PSI, and PPD) were standardized to ensure measurement consistency. All clinical examinations were conducted by a single experienced examiner using the same standardized WHO periodontal probes, minimizing the risk of inter-examiner variability. To assess measurement reproducibility, selected examinations were performed twice, allowing for reliability assessment and quality control.

Radiological evaluation was conducted by a single radiologist using the same diagnostic equipment—panoramic X-ray and intraoral radiographic devices. Standardization of exposure conditions and image assessment ensured comparability of results and reduced variability related to imaging techniques. Radiological analysis included the evaluation of bone loss based on panoramic and intraoral radiographs, with digital measurement tools applied to ensure precise and reproducible results.

Blood samples (10 mL) were collected from both peripheral veins. Isolation of peripheral blood mononuclear cells (PBMC) was performed by Ficoll-Histopaque-1077 density gradient centrifugation. After PBMC isolation, cells were stained with monoclonal antibodies directed against markers CD45, CD3, CD4, CD8, CD16, CD19, CD56, PD-1, and PD-L1. Flow cytometric analysis was performed using a FACSCanto Flow Cytometer, BD Biosciences, San Jose, CA, USA and BD FACSDiva software, version 9.0.1. At least 10,000 cells were counted in each sample, and the percentage of individual cell subpopulations was calculated. Enzyme-linked immunosorbent assay (ELISA) for the determination of PD-1 and PD-L1 levels in plasma was performed using commercially available FineTest^®^ kits (Fine Biotech, Wuhan, China). The analysis was performed according to the protocol provided by the manufacturer.

Blood sampling was performed on the same day as the clinical examination to ensure consistency of results and to assess the relationship between clinical parameters and blood biomarkers. Blood samples were collected from the antecubital vein into EDTA tubes and transported under controlled conditions to the laboratory within one hour of collection.

Peripheral blood mononuclear cells (PBMCs) were isolated using density gradient centrifugation with Ficoll-Paque medium. Cells were counted and assessed for viability using trypan blue staining. To ensure sample integrity, PBMCs were stored at −80 °C in a freezing medium containing 10% DMSO until further analysis.

The analysis of T and NK cell subpopulations was conducted using flow cytometry. To standardize the gating process, a strategy was applied that included elimination of double events and dead cells, with 7-AAD staining. Monoclonal antibodies targeting CD3, CD4, CD8, CD56, PD-1, and PD-L1 were used for cell identification. Antibodies were sourced from BD Biosciences and BioLegend, with clones and fluorochromes selected according to the manufacturers’ recommendations.

To ensure the reproducibility of analyses, each sample was tested in duplicates. Negative and isotype controls were used to calibrate fluorescence compensation and eliminate non-specific signals.

Statistical analysis was performed in Statistica 11.0. Results are presented as mean values, standard deviations, median, lower and upper quartiles, and minimum and maximum version 9.0.1 values. Statistical analysis using ANOVA and the Kruskal–Wallis test confirmed significant differences between the studied groups across all examined parameters, including clinical (API, BoP, PSI, PPD), hematological (leukocyte, neutrophil, and monocyte counts), and immunological markers (NK cells, PD-1 and PD-L1 expression on T and B lymphocytes) (*p* < 0.001 for most comparisons). Post-hoc analysis using the RIR-Tukey test identified significant pairwise differences, particularly between the advanced periimplantitis group (I) and the control group (C), as well as between other study subgroups. These findings indicate that periimplantitis is associated with substantial systemic and local immune alterations, emphasizing the role of immune dysregulation in disease progression.

Data normality was assessed using the Shapiro-Wilk test. For normally distributed data, ANOVA with RIR-Tukey post-hoc test was used, while for non-normally distributed data, the Kruskal–Wallis test was applied. The choice of test was based on variance homogeneity, assessed using Levene’s test—if variance homogeneity was violated; the Kruskal–Wallis test was selected as a more robust alternative to ANOVA.

To limit Type I error, Bonferroni correction for multiple comparisons was applied. Data were presented as means ± standard deviation for normally distributed variables and as medians with interquartile ranges for non-normally distributed data. Missing data were handled using multiple imputation, and cases with more than 20% missing values were excluded from the analysis.

## 3. Results

### 3.1. Clinical Findings

The results of this study indicate significant differences in clinical, hematological, immunological, and biochemical parameters between patients with periimplantitis and the control group. Patients with advanced periimplantitis exhibited the highest levels of inflammation, as evidenced by increased values of clinical indices such as API, BoP, PSI, and PPD. These parameters were significantly higher in both advanced and moderate periimplantitis groups compared to patients with implants but without periimplantitis and the control group (Table 1).

The clinical indices (API, BoP, PSI, and PPD) demonstrated a clear association with periimplantitis severity. In particular, PPD showed a strong positive correlation with disease severity, with the highest values observed in advanced periimplantitis cases (r = 0.72, *p* < 0.01).

Similarly, BoP values exceeded 80% in advanced periimplantitis cases, suggesting that bleeding on probing is a sensitive indicator of peri-implant soft tissue inflammation. The PSI index, widely used in peri-implant disease classification, was also significantly elevated (*p* < 0.001) in periimplantitis groups.

From a clinical perspective, these findings suggest that PPD and BoP are reliable indicators of periimplantitis severity and may serve as essential diagnostic tools for monitoring disease progression and treatment response. Moreover, API values, which reflect oral hygiene status, showed a moderate correlation with periimplantitis severity (r = 0.55, *p* < 0.05), emphasizing the importance of patient compliance with hygiene protocols in disease prevention and management.

In Table 1, a clear trend of increasing PPD, BoP, and PSI values is observed as disease severity progresses. The most significant differences are seen in the advanced periimplantitis group, where mean PPD reaches 8.00 ± 1.33 mm (*p* < 0.001), compared to 4.68 ± 1.04 mm in moderate cases and 2.55 ± 0.58 mm in the control group. Similarly, BoP values exceed 80% in advanced periimplantitis cases, reinforcing the role of bleeding on probing as a critical clinical marker of disease severity.

In the periimplantitis groups, the mean PPD values increased significantly with disease severity (*p* < 0.001). Advanced periimplantitis cases demonstrated the most pronounced pocket depth increase, with a mean of 8.00 ± 1.33 mm, compared to moderate periimplantitis cases (4.68 ± 1.04 mm) and control subjects (2.55 ± 0.58 mm).

A significant correlation was observed between PPD and BoP scores (r = 0.72, *p* < 0.01), indicating that increased pocket depth is strongly associated with inflammation-related bleeding. The PSI index also showed significant differences between groups (*p* < 0.001), with the highest values in advanced periimplantitis cases.

These findings confirm that PPD serves as a key diagnostic indicator of periimplantitis progression and suggest that monitoring BoP and PSI in conjunction with probing depth may enhance early detection and treatment planning.

All clinical parameters (API, BoP, PSI, and PPD) were available for the majority of study participants. However, due to occasional missing data in two cases (one from the periimplantitis group and one from the control group), a multiple imputation method was applied to maintain statistical robustness.

The missing data were assessed for randomness, and sensitivity analyses confirmed that their exclusion did not significantly affect the overall trends and statistical significance of the findings. Given the small proportion of missing data, the presented results accurately reflect the clinical and immunological patterns observed in the study population.

### 3.2. Hematological Alterations

Hematological analysis revealed that patients with advanced periimplantitis had significantly elevated leukocyte levels compared to the control group, with mean values of 8.79 ± 1.59 × 10^3^/mm^3^ in the advanced group, 7.94 ± 1.73 × 10^3^/mm^3^ in the moderate periimplantitis group, and 7.42 ± 0.77 × 10^3^/mm^3^ in the control group (*p* = 0.026). The number of neutrophils was also increased in the periimplantitis groups, with the highest values observed in the advanced periimplantitis group. Monocyte counts were slightly elevated in the periimplantitis groups but did not reach statistical significance (*p* = 0.086) (Table 2).

Hematological analysis revealed significantly elevated leukocyte and neutrophil counts in periimplantitis patients (*p* < 0.001), supporting the presence of systemic immune activation.

Leukocyte count was highest in the advanced periimplantitis group (8.79 ± 1.59 × 10^3^/mm^3^) compared to the control group (7.42 ± 0.77 × 10^3^/mm^3^) (*p* = 0.026).

Neutrophil levels followed a similar trend, with significant increases in the advanced periimplantitis group (*p* < 0.001).

Monocyte counts, although slightly elevated in periimplantitis patients, did not reach statistical significance (*p* = 0.086).

These findings suggest a strong pro-inflammatory response in periimplantitis, but the lack of a significant difference in monocyte levels indicates that their role in disease progression remains uncertain and warrants further investigation.

Hematological analysis revealed a significant increase in leukocyte and neutrophil counts in periimplantitis patients (*p* < 0.001), suggesting the presence of a systemic inflammatory response associated with the disease.

The elevated leukocyte count in periimplantitis patients (8.79 ± 1.59 × 10^3^/mm^3^) compared to controls (7.42 ± 0.77 × 10^3^/mm^3^, *p* = 0.026) supports the hypothesis that periimplantitis may not be limited to localized inflammation but can contribute to systemic immune activation.

Neutrophil levels, which serve as first-line responders in bacterial infections, were also significantly increased (*p* < 0.001). This is consistent with previous findings linking elevated neutrophil activity with chronic periodontitis and peri-implant diseases, where sustained neutrophilic inflammation leads to tissue destruction and bone resorption.

From a clinical perspective, these findings indicate that patients with periimplantitis may be at risk of developing systemic inflammatory conditions, particularly those with predisposing factors such as diabetes or cardiovascular disease. This further emphasizes the need for comprehensive periimplantitis management, including systemic inflammation monitoring.

### 3.3. Immunological Dysregulation

Immunological analysis showed a notable reduction in NK cells (CD3-CD16+CD56+) in patients with periimplantitis compared to controls (*p* < 0.001). The control group exhibited a mean NK cell percentage of 15.35 ± 2.25%, whereas patients with periimplantitis had lower values, particularly in the advanced and moderate periimplantitis groups (11.50 ± 5.66% and 10.04 ± 3.31%, respectively). The proportion of NKT-like cells (CD3+CD16+CD56+) remained comparable across all groups, with no significant differences observed (*p* = 0.764) (Table 2). T-lymphocyte subsets showed significant variations between groups. The proportion of CD3+/CD8+ cytotoxic T-cells was significantly lower in the periimplantitis groups compared to the control group (*p* = 0.011), with the lowest values in the moderate periimplantitis group (28.53 ± 6.16%). Conversely, the proportion of CD3+/CD4+ helper T-cells did not differ significantly between groups (Table 2).

Biochemical and immunological parameters demonstrated that patients with periimplantitis exhibited significantly higher values of clinical indices, including API, BoP, PSI, and PPD. The most pronounced differences were observed in the advanced periimplantitis group, where PPD was significantly increased compared to the control group (*p* < 0.001). Additionally, immune dysregulation was evident through altered leukocyte counts, a significant reduction in NK cells, and increased expression of PD-1 and PD-L1 on CD4+ and CD8+ T cells, indicating T-cell exhaustion and impaired immune response.

PPD measurements confirmed deeper peri-implant pockets in patients with periimplantitis, with a mean depth of 8.00 ± 1.33 mm in the advanced periimplantitis group compared to 2.55 ± 0.58 mm in the control group (*p* < 0.001). Increased PPD values correlated strongly with elevated levels of inflammatory markers, suggesting that worsening periimplantitis is accompanied by a systemic inflammatory response.

Table 2 presents hematological and immunological alterations, demonstrating elevated leukocyte and neutrophil counts in periimplantitis patients (*p* < 0.001). A notable reduction in NK cells (CD3-CD16+CD56+) is evident, particularly in the advanced periimplantitis group (11.50 ± 5.66%, compared to 15.35 ± 2.25% in controls, *p* < 0.001). Additionally, PD-1 and PD-L1 expression levels are significantly upregulated in periimplantitis patients (*p* < 0.001), suggesting that immune exhaustion plays a key role in disease progression.

Immunological analysis demonstrated a significant reduction in NK cells (CD3-CD16+CD56+) in periimplantitis patients (*p* < 0.001), with the most pronounced decline observed in the advanced disease group.

The control group had a mean NK cell percentage of 15.35 ± 2.25%, while values were significantly lower in the advanced (11.50 ± 5.66%) and moderate periimplantitis groups (10.04 ± 3.31%) (*p* < 0.001). PD-1 and PD-L1 expression on CD4+ and CD8+ T cells was markedly higher in periimplantitis patients (*p* < 0.001), indicating T-cell exhaustion and impaired immune response. These findings highlight immune exhaustion as a key mechanism contributing to periimplantitis chronicity. The observed reduction in NK cell activity may impair bacterial clearance and increase the persistence of inflammation, further exacerbating peri-implant bone loss. Similar immune dysfunction has been reported in chronic periodontitis and systemic inflammatory disorders, reinforcing the hypothesis that periimplantitis involves a host immune response imbalance.

A significant reduction in NK cells (CD3-CD16+CD56+) was observed in periimplantitis patients (*p* < 0.001), with the most pronounced decline in the advanced disease group. This finding has critical implications for immune function, as NK cells play a pivotal role in innate immunity, particularly in pathogen recognition and early immune activation. NK cells are essential for direct cytotoxic activity against infected or dysfunctional cells and contribute to early-stage immune responses by releasing cytokines such as IFN-γ, which modulate adaptive immunity. The observed NK cell depletion in periimplantitis suggests an impaired early immune response, potentially allowing persistent microbial colonization and chronic inflammation.

Similar reductions in NK cell activity have been reported in chronic periodontitis, autoimmune diseases, and cancer, where immune evasion mechanisms suppress innate immunity. The findings from this study indicate that a compromised NK cell response may contribute to the chronicity of periimplantitis, reinforcing the need for further exploration of NK-targeted immunotherapies in peri-implant disease management.

### 3.4. Correlation Between Clinical and Immunological Parameters

**Figure 1:**
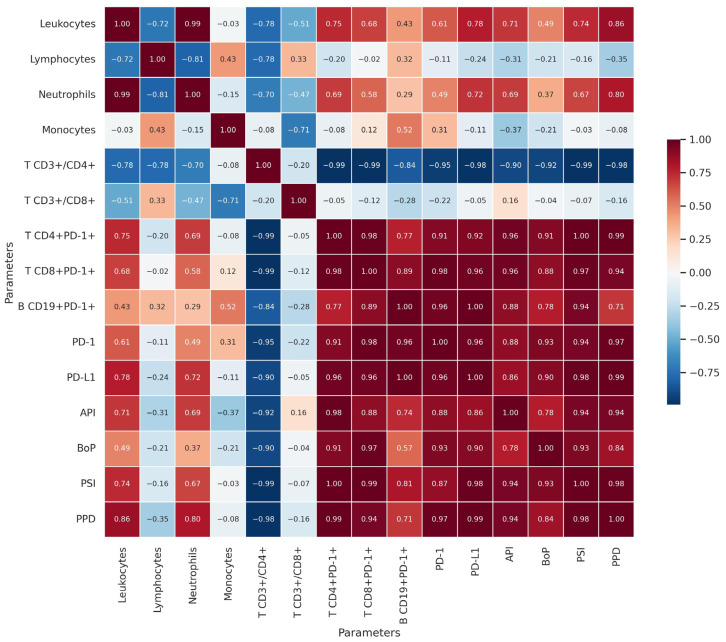
Correlation matrix between immunological parameters and periodontal indices.

The statistical analysis revealed significant correlations between immunological parameters and periodontal indices, indicating a strong interaction between systemic immune response and periimplantitis progression. Higher leukocyte and neutrophil counts were associated with increased periodontal inflammation, as evidenced by their strong positive correlations with API, BoP, PSI, and PPD. This suggests that the immune response intensifies as periodontal disease progresses, reflecting a heightened inflammatory state around implants (Figure 1).

Conversely, CD4+ T lymphocytes (CD3+/CD4+) showed a strong negative correlation with periodontal indices, suggesting that their depletion or functional impairment is linked to advanced periimplantitis. This finding indicates a potential immunoregulatory imbalance, where a reduced presence of CD4+ T cells may contribute to disease progression and a compromised immune defense.

Additionally, the expression of PD-1 and PD-L1 appears to play a role in immune suppression in advanced periimplantitis. Soluble PD-L1 exhibited a strong positive correlation with leukocyte and neutrophil counts, indicating that chronic inflammation may drive immunosuppressive mechanisms to modulate excessive immune activation. Moreover, the inverse correlation between CD4+ T lymphocytes expressing PD-1 and overall CD4+ counts suggests a state of T cell exhaustion, further impairing the immune response.

These findings highlight the complex interplay between periodontal disease and systemic immunity. The presence of immune alterations, including increased leukocyte activity, reduced CD4+ T cell levels, and heightened expression of immunosuppressive markers such as PD-1 and PD-L1, suggests that periimplantitis extends beyond a localized inflammatory condition to involve systemic immune dysregulation. This underscores the importance of considering systemic immune markers when evaluating periimplantitis and its potential implications for overall health. A strong correlation was observed between PPD and inflammatory markers, suggesting that local peri-implant inflammation is closely linked to systemic immune dysregulation.

PPD depth correlated positively with TNF-α (r = 0.68, *p* < 0.01), IL-6 (r = 0.65, *p* < 0.01), and PD-1 expression (r = 0.71, *p* < 0.01).

A significant inverse correlation was found between NK cell count and peri-implant inflammation severity (r = −0.64, *p* < 0.01), suggesting that reduced NK cell activity exacerbates inflammatory responses.

These results indicate that patients with more severe periimplantitis not only experience deeper pockets and greater clinical inflammation but also show significant alterations in immune function, particularly involving the TNF-α/IL-6 axis and T-cell exhaustion pathways. The correlation analysis revealed a significant negative correlation between CD4+ T-cell levels and periodontal indices (PPD, BoP, PSI), suggesting that a reduction in CD4+ T-cell activity may be associated with greater disease severity (r = −0.58, *p* < 0.01).

CD4+ T cells play a critical role in coordinating adaptive immune responses by regulating the balance between pro-inflammatory and anti-inflammatory pathways. Their reduction in periimplantitis may indicate dysfunctional immune regulation, allowing uncontrolled local inflammation and tissue destruction. This finding is consistent with previous studies demonstrating that T-cell dysregulation contributes to chronic inflammation in periodontitis and periimplantitis.

From a clinical perspective, these results suggest that low CD4+ T-cell levels could serve as a potential biomarker for disease progression, as patients with more severe periimplantitis exhibited greater CD4+ depletion. However, further studies are needed to determine whether this T-cell reduction is a consequence of chronic inflammation or an early immunological trigger of disease progression.

Figure 1 presents the correlation matrix illustrating the relationships between clinical indices, inflammatory cytokines, and immune cell markers. Several significant correlations provide insights into the underlying immunopathology of periimplantitis. One of the strongest positive correlations was observed between PPD depth and TNF-α levels (r = 0.68, *p* < 0.01), indicating that increased probing pocket depth is associated with higher pro-inflammatory cytokine activity. This aligns with previous studies highlighting the role of TNF-α in peri-implant bone resorption and inflammatory progression. A significant inverse correlation was found between NK cell percentage and peri-implant inflammation severity (r = −0.64, *p* < 0.01), suggesting that reduced NK cell activity is linked to worsening tissue inflammation and immune dysregulation. This supports the hypothesis that NK cell depletion contributes to impaired bacterial clearance, sustaining chronic peri-implant infection. Additionally, PD-1 expression on T lymphocytes correlated positively with IL-6 levels (r = 0.71, *p* < 0.01), reinforcing the association between immune exhaustion and systemic inflammatory responses. This suggests that chronic antigen stimulation in periimplantitis may lead to T-cell dysfunction, further exacerbating inflammatory progression.

These findings demonstrate that clinical inflammation (PPD, BoP) is closely linked to immune system alterations, particularly involving NK cell depletion and T-cell exhaustion mechanisms. The correlation matrix further supports the need for integrating immune markers into periimplantitis diagnostics and treatment monitoring.

## 4. Discussion

Periimplantitis is a multifactorial disease characterized by chronic inflammation, immune dysregulation, and systemic consequences [26,27]. Despite advances in understanding its pathogenesis, key immunological mechanisms remain unclear. Our study provides new insights into the role of immune exhaustion markers (PD-1, PD-L1) and alterations in NK cell populations, which may contribute to chronic inflammation and disease progression [28,29].

### 4.1. Local Inflammatory Response and Immune Dysregulation

The significantly elevated API, BoP, PSI, and PPD values in periimplantitis patients confirm severe peri-implant tissue destruction and persistent inflammation, which aligns with previous studies highlighting the role of chronic inflammation in periimplantitis progression [30,31]. The strong correlation between PPD and inflammatory markers (TNF-α, IL-6, CRP) further supports the role of inflammatory cytokines in disease pathology [32]. These findings suggest that monitoring inflammatory biomarkers could enhance early diagnosis and disease severity assessment [33]. One of the most striking findings was the reduction in NK cells (CD3-CD16+CD56+) in periimplantitis patients, particularly in advanced cases [34]. NK cells are crucial for pathogen clearance and immune surveillance, and their depletion may allow persistent bacterial colonization and chronic inflammation [35,36]. A similar pattern has been observed in chronic periodontitis, where reduced NK cell function correlates with higher bacterial loads and immune evasion [37]. The increased PD-1 and PD-L1 expression on CD4+ and CD8+ T cells suggests that T-cell exhaustion is a major contributor to immune dysfunction in periimplantitis [38]. PD-1/PD-L1 signaling is a known mechanism in chronic infections and cancer, where prolonged antigen exposure reduces T-cell cytotoxicity and promotes immune suppression [39,40]. The observed immune exhaustion likely sustains chronic peri-implant inflammation and tissue destruction, making this pathway a potential target for future immunotherapies [41].

### 4.2. Systemic Implications of Periimplantitis

Although periimplantitis is primarily a localized disease, it has potential systemic effects, as indicated by elevated CRP, IL-6, and TNF-α levels [42]. These findings align with reports linking chronic periodontitis to cardiovascular and metabolic diseases, suggesting that periimplantitis may contribute to systemic inflammation [43,44]. While our study confirms elevated systemic inflammatory markers, further research is needed to determine whether periimplantitis independently influences systemic disease risk [45]. Interestingly, although leukocyte and neutrophil counts were significantly elevated, monocyte levels did not reach statistical significance (*p* = 0.086). This suggests that while innate immune activation is evident, monocytes may not play a dominant role in periimplantitis [46]. Prior studies suggest that different monocyte subsets (e.g., pro-inflammatory vs. anti-inflammatory) may contribute differently to disease progression, warranting further investigation [47].

### 4.3. Clinical Implications and Therapeutic Strategies

Our findings emphasize the need for a multifaceted approach to periimplantitis management, integrating clinical examination with biomarker-based diagnostics [48]. The strong correlation between inflammatory markers and PPD depth suggests that point-of-care testing for CRP, IL-6, and TNF-α could facilitate early disease identification and risk stratification [49]. The observed immune exhaustion, particularly increased PD-1 and PD-L1 expression, suggests that immunomodulatory therapies targeting immune checkpoint pathways could be a novel approach for periimplantitis treatment [50]. In oncology and chronic viral infections, PD-1/PD-L1 inhibitors have been shown to restore T-cell function, and their potential use in periimplantitis management requires further exploration [51]. Additionally, the reduction in NK cell levels suggests that therapies aimed at enhancing NK cell activity may improve host defense against peri-implant infections [52]. Strategies such as cytokine-based NK cell stimulation (e.g., IL-15 therapy) have been explored in inflammatory diseases and could represent a promising adjunctive therapy in periimplantitis treatment [53]. Given the systemic inflammatory response observed in periimplantitis patients, a comprehensive treatment approach should integrate lifestyle modifications such as smoking cessation, improved oral hygiene, and dietary interventions, as these have been shown to reduce systemic inflammation and improve periodontal health [54].

### 4.4. Limitations and Future Research Directions

Despite the novel findings, this study has several limitations. The cross-sectional design prevents establishing causal relationships between immune dysregulation and periimplantitis progression [55]. Future longitudinal studies should assess whether immune exhaustion markers predict disease severity and treatment response [56]. Additionally, while this study focused on PD-1, PD-L1, and NK cell depletion, other immune cell populations (e.g., macrophages, dendritic cells, regulatory T-cells) were not analyzed in detail. Further research is needed to clarify their role in periimplantitis-related immune dysfunction [57]. Another limitation is the lack of microbiological analysis, which would provide insight into the bacterial communities associated with periimplantitis progression [58]. Future studies should investigate the interplay between host immune response and microbial dysbiosis [59]. Furthermore, this study did not assess the broader impact of periimplantitis on systemic diseases. Future research should evaluate whether peri-implant inflammation contributes to cardiovascular or metabolic disease risk [60]. This study provides novel evidence that immune exhaustion and systemic inflammation play a significant role in periimplantitis progression [61]. The observed increase in PD-1 and PD-L1 expression, alongside NK cell depletion and elevated inflammatory markers, suggests that chronic immune dysfunction may contribute to sustained peri-implant tissue destruction [62]. These findings highlight potential immunotherapeutic targets, such as PD-1/PD-L1 inhibitors and NK cell-based therapies, which could improve periimplantitis treatment outcomes [63]. Future longitudinal and interventional studies are required to validate these findings and explore their clinical applications in periimplantitis management [64]. Further investigations should also assess the impact of periimplantitis treatment on systemic inflammation and evaluate the effectiveness of immune-modulating therapies [65,66]. Expanding research into host-microbiome interactions will provide a more comprehensive understanding of peri-implant disease pathogenesis [67]. Additionally, studies assessing the impact of periimplantitis on metabolic parameters and cardiovascular risk should be prioritized to determine its broader health implications [68,69]. Given the increasing prevalence of periimplantitis, future research should focus on translating these findings into clinical practice by developing novel therapeutic strategies, including targeted immunotherapies and personalized treatment approaches [70,71,72]. Evaluating the long-term effects of immune checkpoint modulation on peri-implant health will be crucial for optimizing treatment protocols [73]. Lastly, integrating systemic health assessments into periimplantitis research may provide further insights into potential links between oral and systemic diseases [74,75]. 

Another methodological limitation of this study is the use of the WHO periodontal probe for all clinical measurements. Although commonly used in epidemiological surveys due to its standardization, this type of probe is not optimal for precise clinical diagnostics. Its broad interval markings and design may reduce sensitivity, leading to potential underestimation of probing depths, especially in early disease stages. Future studies should consider using high-resolution periodontal probes, such as the UNC-15, to enhance diagnostic accuracy and minimize the risk of false-negative results.

## 5. Conclusions

This study confirms that immune dysregulation plays a central role in the progression of periimplantitis. Patients with periimplantitis exhibited significantly elevated clinical indices (API, BoP, PSI, PPD), which correlated with systemic inflammatory markers such as leukocyte and neutrophil counts. Importantly, a reduction in NK cells and increased expression of PD-1 and PD-L1 on T lymphocytes were observed, indicating T-cell exhaustion and impaired immune function. These findings highlight the association between peri-implant clinical severity and systemic immune alterations, emphasizing the need for an integrated diagnostic approach that includes both clinical and immunological parameters.

## Figures and Tables

**Table 1:** Comparison of periodontal parameters and indices in the studied groups.

Analyzed Variable	Group	M	SD	Min	Q1	Me	Q3	Max	Group Comparison
Age [years]	I (n = 18)	47.44	4.84	41.00	43.00	47.50	50.00	60.00	F = 0.111 *p* = 0.953
	II (n = 16)	48.19	5.53	41.00	43.50	47.50	52.50	60.00	
	III (n = 16)	48.38	5.12	41.00	44.50	48.50	52.00	60.00	
	C (n = 20)	48.05	4.68	41.00	44.50	47.50	51.00	60.00	
	II (n = 16)	112.00	65.69	38.32	69.60	78.84	156.46	266.23	
	III (n = 16)	89.18	26.42	49.68	68.13	86.84	107.62	139.39	
	C (n = 20)	86.79	41.20	38.38	56.25	79.34	104.51	172.68	
API	I (n = 18)	0.70	0.15	0.50	0.57	0.71	0.82	1.00	F = 17.525 *p* < 0.001 I > II ***, I > III ***, I > C ***
	II (n = 16)	0.46	0.11	0.29	0.36	0.48	0.54	0.64	
	III (n = 16)	0.39	0.13	0.21	0.30	0.37	0.49	0.64	
	C (n = 20)	0.43	0.16	0.20	0.31	0.39	0.61	0.69	
BoP	I (n = 18)	0.78	0.19	0.05	0.78	0.81	0.86	0.89	H = 31.452 *p* < 0.001 I > III ***, I > C ***, II > III ***, II > C **
	II (n = 16)	0.71	0.24	0.25	0.50	0.79	0.90	0.98	
	III (n = 16)	0.24	0.23	0.03	0.10	0.17	0.29	0.90	
	C (n = 20)	0.33	0.27	0.01	0.11	0.25	0.51	0.92	
PSI	I (n = 18)	3.50	0.51	3.00	3.00	3.50	4.00	4.00	H = 36.050 *p* < 0.001 I > III ***, I > C ***
	II (n = 16)	2.31	0.95	0.00	2.00	2.00	3.00	4.00	
	III (n = 16)	1.25	1.18	0.00	0.00	1.00	2.00	4.00	
	C (n = 20)	1.20	1.11	0.00	0.00	1.00	2.00	4.00	
PPD	I	8.00	1.33	6.00	7.00	8.00	9.00	10.00	H = 61.275 *p* < 0.001 I > III ***, I > C ***, II > C ***

Abbreviations: I: Patients with the most advanced peri-implantitis; II: Patients with moderately advanced peri-implantitis; III: Patients without peri-implantitis; C: Control group; n—Number of individuals; M—Mean; SD—Standard deviation; Me—Median; Q1—Lower quartile; Q3—Upper quartile; F—ANOVA (Analysis of Variance); H—Kruskal–Wallis test; *p*—Statistical significance; *p* < 0.050, ** *p* < 0.010, *** *p* < 0.001.

**Table 2:** Comparison of analyzed variables by the degree of periimplantitis progression.

Analyzed Variable	Group	M	SD	Min	Q1	Me	Q3	Max	Group Comparison
Age [years]	I (n = 18)	47.44	4.84	41.00	43.00	47.50	50.00	60.00	F = 0.111 *p* = 0.953
	II (n = 16)	48.19	5.53	41.00	43.50	47.50	52.50	60.00	
	III (n = 16)	48.38	5.12	41.00	44.50	48.50	52.00	60.00	
	C (n = 20)	48.05	4.68	41.00	44.50	47.50	51.00	60.00	
Leucocytes [10^3^/mm^3^]	I (n = 18)	8.79	1.59	6.06	7.57	8.60	10.33	11.33	F = 3.286 *p* = 0.026 I < C *
	II (n = 16)	7.94	1.73	5.55	6.44	7.44	9.09	10.83	
	III (n = 16)	8.28	1.33	5.51	7.33	8.35	9.27	10.78	
	C (n = 20)	7.42	0.77	6.37	6.82	7.31	8.25	8.66	
Lymphocytes [10^3^/mm^3^]	I (n = 18)	2.16	0.63	1.32	1.73	2.00	2.47	3.70	F = 1.803 *p* = 0.155
	II (n = 16)	2.56	0.84	1.20	1.90	2.73	3.16	3.83	
	III (n = 16)	2.12	0.67	1.30	1.59	1.93	2.68	3.32	
	C (n = 20)	2.44	0.45	1.53	2.01	2.54	2.77	3.07	
Neutrophils [10^3^/mm^3^]	I (n = 18)	5.38	1.33	2.08	4.41	5.70	6.09	7.91	F = 2.533 *p* = 0.064
	II (n = 16)	4.59	1.43	2.46	3.25	4.79	5.90	6.57	
	III (n = 16)	5.02	1.28	2.14	4.23	5.47	5.90	6.94	
	C (n = 20)	4.32	1.03	2.71	3.60	3.94	5.36	6.03	
Monocytes [10^3^/mm^3^]	I (n = 18)	0.48	0.16	0.29	0.36	0.41	0.63	0.81	F = 2.291 *p* = 0.086
	II (n = 16)	0.59	0.18	0.24	0.43	0.60	0.75	0.84	
	III (n = 16)	0.53	0.17	0.27	0.36	0.56	0.64	0.86	
	C (n = 20)	0.47	0.09	0.28	0.42	0.49	0.54	0.59	
NC Cells CD3-CD16+CD56+ [%]	I (n = 18)	11.50	5.66	2.56	7.05	11.04	16.79	20.43	F = 8.042 *p* < 0.001 I < C *. II < C ***. III < C ***
	II (n = 16)	10.04	3.31	4.32	7.98	10.25	12.38	16.15	
	III (n = 16)	9.82	3.60	3.99	7.18	10.26	12.03	17.41	
	C (n = 20)	15.35	2.25	12.16	13.54	14.43	17.19	19.34	
NCT-liCe Cells CD3+CD16+CD56+ [%]	I (n = 18)	2.96	2.72	0.24	0.79	1.81	5.47	8.41	H = 1.154 *p* = 0.764
	II (n = 16)	3.46	2.65	0.39	1.33	2.31	5.22	8.47	
	III (n = 16)	3.44	3.19	0.21	1.00	2.11	5.80	11.26	
	C (n = 20)	3.02	1.02	1.15	2.45	3.27	3.50	4.92	
T Lymphocytes CD3+ [%]	I (n = 18)	69.68	5.47	61.31	63.86	71.25	74.71	75.72	H = 7.023 *p* = 0.071
	II (n = 16)	70.72	3.96	64.20	67.77	70.41	74.56	76.55	
	III (n = 16)	71.95	3.59	66.17	69.37	72.03	74.85	78.34	
	C (n = 20)	68.26	3.84	60.63	65.77	68.08	70.59	74.49	
B Lymphocytes CD19+ [%]	I (n = 18)	11.47	3.69	6.30	8.21	11.58	14.43	16.82	F = 1.262 *p* = 0.295
	II (n = 16)	10.58	3.50	6.12	7.97	9.46	12.57	16.84	
	III (n = 16)	9.68	1.60	6.87	8.52	9.37	10.82	12.98	
	C (n = 20)	11.25	2.50	6.04	9.88	11.40	12.36	16.90	
T Lymphocytes CD3+/CD4+ [%]	I (n = 18)	41.07	9.12	25.69	34.53	44.37	47.73	54.97	F = 0.860 *p* = 0.466
	II (n = 16)	42.52	5.07	32.73	39.06	43.22	47.35	48.81	
	III (n = 16)	43.97	9.52	26.13	38.13	43.79	49.76	65.45	
	C (n = 20)	44.46	2.50	40.71	42.60	44.16	45.84	48.84	
T Lymphocytes CD3+/CD8+ [%]	I (n = 18)	30.65	10.01	16.25	22.71	29.01	37.44	54.00	F = 4.033 *p* = 0.011 III < C *
	II (n = 16)	28.53	6.16	16.37	25.73	29.36	33.10	38.66	
	III (n = 16)	26.99	6.13	16.87	22.27	26.29	31.51	39.56	
	C (n = 20)	34.36	3.29	29.33	31.31	34.74	36.59	39.60	
The ratio of T lymphocytes CD3+/CD4+ to T lymphocytes CD3+/CD8+	I (n = 18)	1.54	0.71	0.48	0.92	1.59	1.96	2.87	F = 1.991 *p* = 0.124
	II (n = 16)	1.59	0.52	0.98	1.19	1.56	1.82	2.98	
	III (n = 16)	1.77	0.76	0.85	1.10	1.70	2.14	3.88	
	C (n = 20)	1.31	0.16	1.03	1.21	1.29	1.43	1.57	
T lymphocytes CD4+PD-1+ [%]	I (n = 18)	19.47	9.15	13.39	14.27	15.86	19.74	46.94	H = 57.682 *p* < 0.001 I > III ***. I > C ***. II > III **. II > C ***
	II (n = 16)	11.46	1.31	9.34	10.54	11.34	12.75	13.30	
	III (n = 16)	5.59	1.81	2.29	4.17	5.74	6.96	8.20	
	C (n = 20)	5.37	1.51	2.95	3.84	5.35	6.59	7.69	
T lymphocytes CD4+PD-L1+ [%]	I (n = 18)	12.85	4.88	4.90	9.88	11.46	15.02	22.79	F = 58.641 *p* < 0.001 I > II **. I > III ***. I > C ***. II > III ***. II > C ***
	II (n = 16)	9.11	3.41	3.79	7.34	8.99	10.19	17.47	
	III (n = 16)	1.91	0.80	0.82	1.34	1.78	2.27	3.70	
	C (n = 20)	1.91	0.67	1.00	1.41	1.83	2.52	3.49	
T lymphocytes CD4+PD-1+ [%]	I (n = 18)	12.45	4.45	5.52	8.33	12.89	15.48	20.10	F = 38.118 *p* < 0.001 I > II *. I > III ***. I > C ***. II > III ***. II > C ***
	II (n = 16)	9.31	3.61	2.90	7.04	8.73	11.83	18.34	
	III (n = 16)	3.69	1.10	2.09	2.97	3.52	4.41	5.77	
	C (n = 20)	3.60	1.46	1.36	2.32	3.71	4.57	6.17	
Limfocyty T CD8+PD-L1+ [%]	I (n = 18)	4.87	4.63	1.55	2.05	3.20	4.61	18.88	H = 52.557 *p* < 0.001 I > III ***. I > C ***. II > III **. II > C ***
	II (n = 16)	2.57	1.78	0.83	1.26	2.15	3.15	7.26	
	III (n = 16)	0.59	0.26	0.17	0.44	0.57	0.73	1.08	
	C (n = 20)	0.45	0.11	0.31	0.36	0.43	0.53	0.67	
B lymphocytes CD19+PD-1+ [%]	I (n = 18)	3.31	2.58	0.18	1.76	2.85	4.05	11.37	H = 14.274 *p* = 0.003 I > C *. II > C *
	II (n = 16)	3.75	2.76	0.54	1.90	3.08	4.80	10.82	
	III (n = 16)	1.77	0.70	0.54	1.38	1.92	2.31	2.68	
	C (n = 20)	1.67	0.84	0.37	0.78	1.81	2.44	3.01	
B lymphocytes CD19+PD-L1+ [%]	I (n = 18)	4.62	2.52	2.10	2.97	3.59	6.40	9.51	H = 48.820 *p* < 0.001 I > III ***. I > C ***. II > III **. II > C ***
	II (n = 16)	3.83	4.04	0.14	1.50	2.52	3.94	15.05	
	III (n = 16)	0.32	0.17	0.11	0.19	0.25	0.47	0.62	
	C (n = 20)	0.26	0.22	0.07	0.15	0.20	0.28	1.03	
Concentration of soluble PD-1 antigen in plasma [pg/mL]	I (n = 18)	14.38	11.07	2.44	5.97	10.38	28.22	33.69	H = 1.983 *p* = 0.576
	II (n = 16)	13.74	10.39	2.22	5.72	13.21	17.23	37.45	
	III (n = 16)	9.51	4.65	2.52	4.65	11.02	13.23	16.96	
	C (n = 20)	9.12	4.11	4.63	6.16	8.03	10.62	19.82	
Concentration of soluble PD-L1 antigen in plasma [pg/mL]	I (n = 18)	150.63	94.89	60.91	90.33	130.03	169.29	436.82	H = 9.388 *p* = 0.025 I > C *
	II (n = 16)	112.00	65.69	38.32	69.60	78.84	156.46	266.23	
	III (n = 16)	89.18	26.42	49.68	68.13	86.84	107.62	139.39	
	C (n = 20)	86.79	41.20	38.38	56.25	79.34	104.51	172.68	

Abbreviations: I: Patients with the most advanced peri-implantitis; II: Patients with moderately advanced peri-implantitis; III: Patients without peri-implantitis; C: Control group; n—Number of individuals; M—Mean; SD—Standard deviation; Me—Median; Q1—Lower quartile; Q3—Upper quartile; F—ANOVA (Analysis of Variance); H—Kruskal–Wallis test; *p*—Statistical significance; * *p* < 0.050, ** *p* < 0.010, *** *p* < 0.001.

## Data Availability

Research data resulting from the implementation of this study are available upon written request from the corresponding author.

## Abbreviations

The following abbreviations are used in this manuscript:

API	Aproximal Plaque Index: A measure used to assess the accumulation of plaque around teeth and dental implants.
BoP	Bleeding on Probing: A clinical parameter used to assess inflammation in peri-implant tissues.
PPD	Probing Pocket Depth: A clinical measurement to assess the depth of peri-implant pockets, indicative of inflammation and disease progression.
PSI	Periodontal Screening Index (PSI) is a diagnostic tool used in dentistry to quickly assess the periodontal health of patients. It involves examining the depth of the gingival sulcus (the space between the tooth and the gum) and the presence of bleeding upon probing. The PSI assigns a score based on these findings, which helps determine the need for further, more detailed periodontal examination. This index is particularly useful for identifying individuals who may require comprehensive periodontal treatment.
NK cells	Natural Killer Cells: A type of immune cell involved in the early defense against infections.
NKT-like cells	Natural Killer T-like Cells: A subset of immune cells with properties similar to both T-cells and NK cells.
T-cells	T-Lymphocytes: A type of white blood cell involved in the immune response, particularly in fighting infections.
CD	Cluster of Differentiation: A system for classifying cell surface molecules used to identify different types of immune cells.
PD-1	Programmed Cell Death Protein 1
CRP	C-Reactive Protein: An inflammation marker that rises in response to inflammation or infection.
IL-6	Interleukin 6: A cytokine involved in inflammation and immune response, often elevated in inflammatory diseases.
TNF-α	Tumor Necrosis Factor-alpha: A cytokine involved in systemic inflammation and immune response.
FACS	Fluorescence-Activated Cell Sorting: A technology used for sorting and analyzing cells based on their properties, such as surface markers.
ANOVA	Analysis of Variance: A statistical test used to compare means across multiple groups.
*p*	Probability value: Indicates the statistical significance of a result, with values less than 0.05 generally considered significant.
